# Supplementary material for: From sequence to enzyme mechanism using multi-label machine learning
Source: BMC Bioinformatics. 2014 May 19;15:150. doi: 10.1186/1471-2105-15-150 (PMC4229970; doi:10.1186/1471-2105-15-150)
Supplement: Additional file 2 — Java code of ml2db. Additional file ml2db_code.tar.gz contains the Java source code to run the multi-label machine learning experiments and save the results to database. The code’s Javadoc is included. [file 1471-2105-15-150-S2.zip › additional file 2/ml2db/utils/doc/index-files/index-24.html]

X-Index


JavaScript is disabled on your browser.


- Overview
- Package
- Class
- Use
- Tree
- Deprecated
- Index
- Help

- Prev Letter
- Next Letter

- Frames
- No Frames

- All Classes

A B C D E F G H I J K L M N O P Q R S T U V W X Y 


## X

xmlElementToString(XmlNode) - Static method in class uk.ac.standrews.utils.main.EntrezUtils
:   Test method to print to string a single Entrez eSummary xml element

XmlNode - Class in uk.ac.standrews.utils.main.webutils.simpledomparser
:   `SimpleElement` is the only node type for simplified DOM model.

XmlNode(String) - Constructor for class uk.ac.standrews.utils.main.webutils.simpledomparser.XmlNode


XmlNodeTest - Class in uk.ac.standrews.utils.test
:   Class

XmlNodeTest() - Constructor for class uk.ac.standrews.utils.test.XmlNodeTest


XmlSearcher - Class in uk.ac.standrews.utils.main.webutils.simpledomparser
:   Executes a search over a SimpleElement (an XML tree)

XmlSearcher() - Constructor for class uk.ac.standrews.utils.main.webutils.simpledomparser.XmlSearcher


XmlSearcherTest - Class in uk.ac.standrews.utils.test
:   Class

XmlSearcherTest() - Constructor for class uk.ac.standrews.utils.test.XmlSearcherTest


XMLUtils - Class in uk.ac.standrews.utils.main.webutils
:   XML utilities.

XMLUtils() - Constructor for class uk.ac.standrews.utils.main.webutils.XMLUtils


XmlUtilsTest - Class in uk.ac.standrews.utils.test
:   Class

XmlUtilsTest() - Constructor for class uk.ac.standrews.utils.test.XmlUtilsTest

A B C D E F G H I J K L M N O P Q R S T U V W X Y

- Overview
- Package
- Class
- Use
- Tree
- Deprecated
- Index
- Help

- Prev Letter
- Next Letter

- Frames
- No Frames

- All Classes
